# A Model for Transport Phenomena in a Cross-Flow Ultrafiltration Module with Microchannels

**DOI:** 10.3390/membranes1010013

**Published:** 2010-12-16

**Authors:** Aiko Nishimoto, Shiro Yoshikawa, Shinichi Ookawara

**Affiliations:** Department of Chemical Engineering Graduate School for Science and Engineering, Tokyo Institute of Technology, Meguro-ku, Tokyo, 152-8550, Japan; E-Mails: aiko_nishimoto@ihi.co.jp (A.N.); sokawara@chemeng.titech.ac.jp (S.O.)

**Keywords:** ultrafiltration, microchannel, cross-flow, transport phenomena, modelling

## Abstract

Cross-flow ultrafiltration of macromolecular solutions in a module with microchannels is expected to have the advantages of fast diffusion from the membrane surface and a high ratio of membrane surface area to feed liquid volume. Cross-flow ultrafiltration modules with microchannels are expected to be used for separation and refining and as membrane reactors in microchemical processes. Though these modules can be applied as a separator connected with a micro-channel reactor or a membrane reactor, there have been few papers on their performance. The purpose of this study was to clarify the relationship between operational conditions and performance of cross-flow ultrafiltration devices with microchannels. In this study, Poly Vinyl Pyrrolidone (PVP) aqueous solution was used as a model solute of macromolecules such as enzymes. Cross-flow ultrafiltration experiments were carried out under constant pressure conditions, varying other operational conditions. The permeate flux decreased in the beginning of each experiment. After enough time passed, the permeate flux reached a constant value. The performance of the module was discussed based on the constant values of the flux. It was observed that the permeate flux increased with increasing transmembrane pressure (*TMP*) and feed flow rate, and decreased with an increase of feed liquid concentration. A model of the transport phenomena in the feed liquid side channel and the permeation through the membrane was developed based on the concentration and velocity distributions in the feed side channel. The experimental results were compared with those based on the model and the performance of the ultrafiltration module is discussed.

## 1. Introduction

Microchannels attract attention as cross flow ultrafiltration modules for concentration or separation processes in micro chemical processes. Such microchannels would be used downstream of micro chemical reactors or as membrane reactors in micro chemical processes. A microchannel has a high aspect ratio and large specific surface area. Cross-flow ultrafiltration modules with microchannels are, therefore, considered to be preferable for membrane separation processes in terms of the ratio of feed liquid volume to membrane area and the short diffusion length from membrane surface to bulk flow region.

There are many papers focused on the model of the permeation process in cross flow ultrafiltration in macro scale modules, in which the permeate flux of ultrafiltration of macromolecular solution is usually analyzed by the gel polarization model [1], osmotic-pressure model [2], or resistance-in-series model [3-4]. The permeate flux in the ultrafiltration process has been estimated based on one of the models or a combination of a number of the models considering the factors which control the filtration process. On the other hand, researches on the ultrafiltration in micro chemical processes are focused mainly on the fabrication of devices. Ikuta *et al.* [5] reported the dead-end ultrafiltration device in an integrated biochemical chip. Martin *et al.* [6] studied the fabrication methods for micro reactors including membrane devices. There are few papers on the permeation processes in the cross-flow ultrafiltration devices with microchannels.

In the authors' previous paper [7], a cross-flow ultrafiltration module with microchannels, which are considered to be used for the filtration of macromolecular solutions, was designed and fabricated. In addition, a model on the transport phenomena based on the concentration polarization in the channel was proposed. By means of the model, the permeate fluxes under various conditions were estimated and the performance of the module was discussed. In the previous paper [7], only one module was fabricated and tested. The dependence of the performance on the length of the channel was not considered. In this study, two types of module with different lengths of channels are fabricated. The modules consist of feed liquid side and permeate liquid side channels. Membrane is put between two channels and experiments of cross flow ultrafiltration are carried out. PolyVinylPyrrolidone (PVP) aqueous solution is used as a model solute of macromolecules such as enzymes. Changes in permeate flux with time are obtained for various conditions. Based on the results, the relation between the permeate flux and the operational conditions, including the length of the channel, are discussed. Furthermore, the previous model [7] is improved, considering the change in the thickness of the concentration polarization layer. In the model, distributions of concentration, velocity, permeate flux, osmotic pressure at the membrane surface and transmembrane pressure in the channel are calculated considering the mass balance, concentration polarization, change in viscosity with concentration and the relationship between osmotic pressure and concentration. As a result, the permeate flux of the module is calculated as an average value of the distribution of permeate flux in the axial direction of the channel. The results are compared with those of experiments and the validity of the model and the relationship among the permeate flux, operational conditions and the length of the channels are discussed.

## 2. Ultrafiltration Module with Microchannels

**Figure 1:**
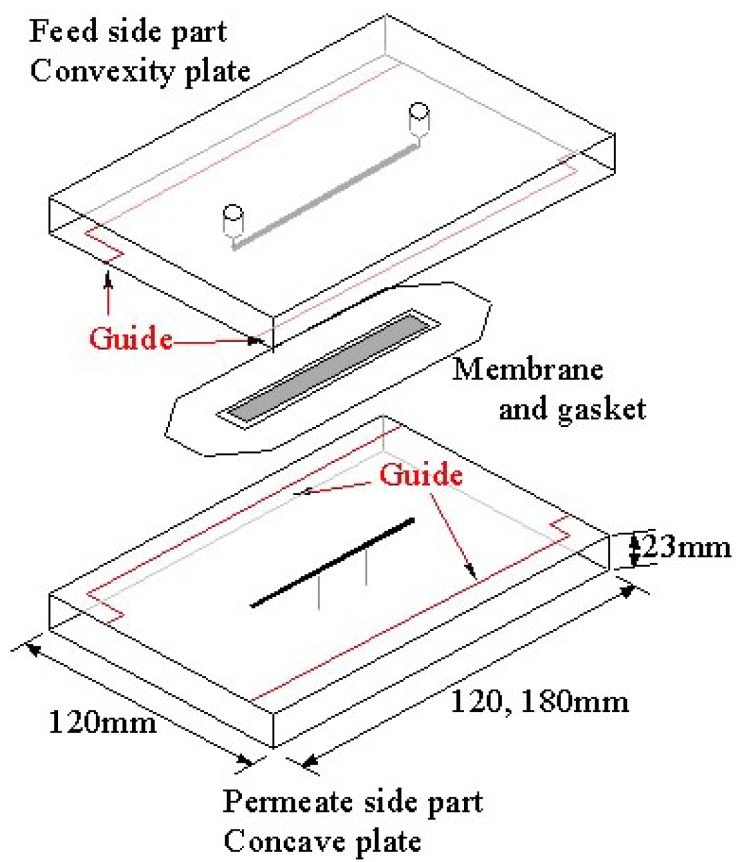
A schematic illustration of a filter module with microchannels.

**Figure 2:**
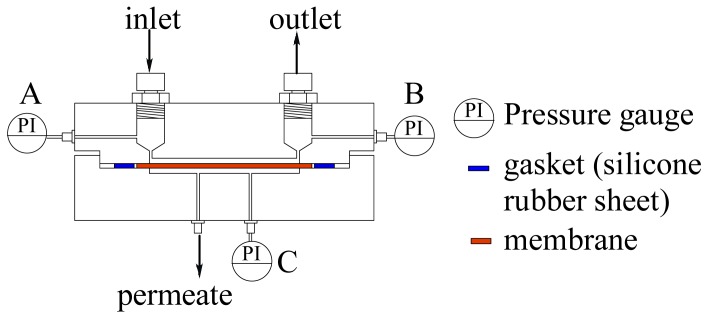
A schematic illustration of a longitudinally cut cross-sectional view of the module.

Figure 1 shows an exploded schematic view of an ultrafiltration module with microchannels. The module consists of two parts made of acrylic transparent resin. The upside part in Figure 1 is the feed liquid side and the lower is the permeate side. These feed and permeate parts, each having a groove with a height of 0.1 mm and a width of 1.5 mm, form microchannels above and below an ultrafiltration membrane by clipping the membrane and gasket. Two types of module with different lengths of channels were used. One has channels of 60 mm and the other has those of 90 mm. The width and thickness of each part are 120 mm and 23 mm, respectively. The lengths are 120 mm and 180 mm for the channel length 60 mm and 90 mm, respectively. The parts are cut by an end mill. Figure 2 shows a longitudinally cut cross-sectional view of the assembled module. As described above, the feed and permeate side channels are formed above and below the membrane, respectively. Measurement by means of three pressure gauges, designated (A), (B) and (C) in the figure, is mentioned in the following section.

## 2. Experimental Methods

Poly Vinyl Pyrrolidone (PVP) aqueous solution was used as a model liquid of a macromolecule solution produced in a micro chemical system. The rheology of various concentrations of PVP aqueous solution was investigated by means of a rheometer (Physica MCR-300 Anton Paar GmbH, Austria). It appeared that the solution behaved as a Newtonian fluid. It was confirmed that the relationship between viscosity *μ* [Pa·s] and concentration *C* [wt %] correlated appropriately in the range of experimental conditions by the following equation [7].

(1) $$\mu =1.22\times 10^{-3}\text{exp}(1.51\times 10^{-1}C)$$

This equation is used in the estimation of permeate flux based on a new model.

A regenerated cellulose membrane with a molecular weight cut-off of 5,000 was used in the experiments. The average molecular weight of PVP is 35,000. It would be, therefore, considered that PVP is almost completely rejected by the membrane.

**Figure 3:**
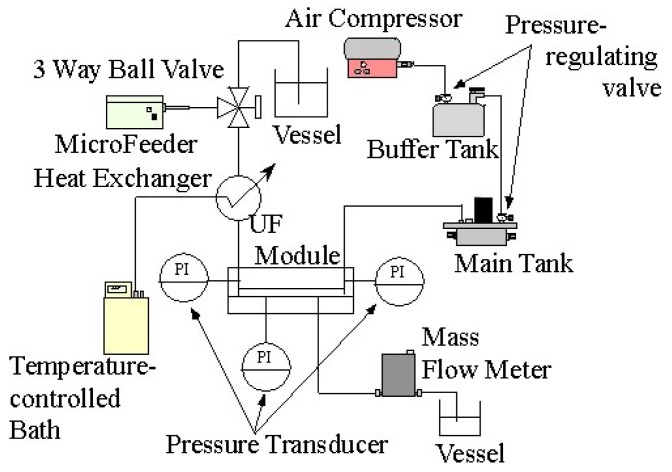
A Schematic illustration of the experimental apparatus.

Figure 3 shows a schematic illustration of the experimental apparatus. Feed liquid was fed by a micro feeder to the inlet of the ultrafiltration module at a constant volume flow rate. The temperature of the feed liquid was kept at 10 degrees Celsius by means of a heat exchanger connected with a temperature controlling bath. The liquid flowed into the feed liquid side channel and was concentrated in the module. Then, it flowed out at the outlet of the feed side channel. Part of the solvent water of the feed liquid permeated through the membrane. It flowed out at the outlet of the permeate side channel. The flow rate of the permeate liquid was measured downstream of the outlet by means of a liquid mass flow sensor (SLG1430-320: Sensirion AG). The feed channel was pressurized by an air compressor by way of a buffer tank with two pressure regulating valves. The pressures of feed and permeate side channels were measured by pressure transducers (PGMC-A-200KP: Kyowadengyo Ltd.) connected to the inlet (A) and outlet (B) of the feed side channel and the permeate side channel (C), as shown in Figure 2. The pressure in the permeate side was assumed to be constant because the flow rate was much smaller than that in the feed side. The transmembrane pressure (*TMP*) was defined as the difference between the average pressure at the inlet and the outlet and pressure in the permeate side channel. *TMP* was kept constant throughout the experiments. The flow rate and concentration of feed liquid, *TMP* and the length of the channels were chosen as parameters of experimental conditions. The ranges of the parameters are shown in Table 1.

**Table 1:** Experimental conditions.

Feed flow rate: *Q*_f_	20, 100 μL·min ^−1^
Feed concentration: *C*_f_	1, 2, 4, 6, 10 wt %
*TMP*	75, 100, 125, 150 kPa
Length of channels: *L*	60, 90 mm

## 3. Experimental Results

As described above, the flow rate of the permeate liquid was measured. The averaged permeate flux over the whole membrane area was calculated by dividing the volume flow rate by the effective membrane area, *i.e.,* 90 and 135 mm^2^ for *L* = 60 mm and 90 mm, respectively. The permeate flux decreased in the beginning of each experiment. After enough time passed, the permeate flux reached a constant value. The performance of the module was discussed based on the constant values.

**Figure 4:**
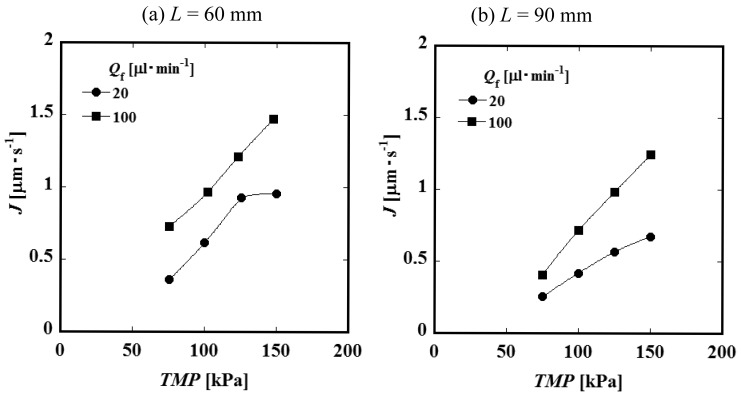
Relationships between permeate flux and *TMP* (*C*_f_ = 10 wt %) when (**a**) *L* = 60 mm and (**b**) *L* = 90 mm.

**Figure 5:**
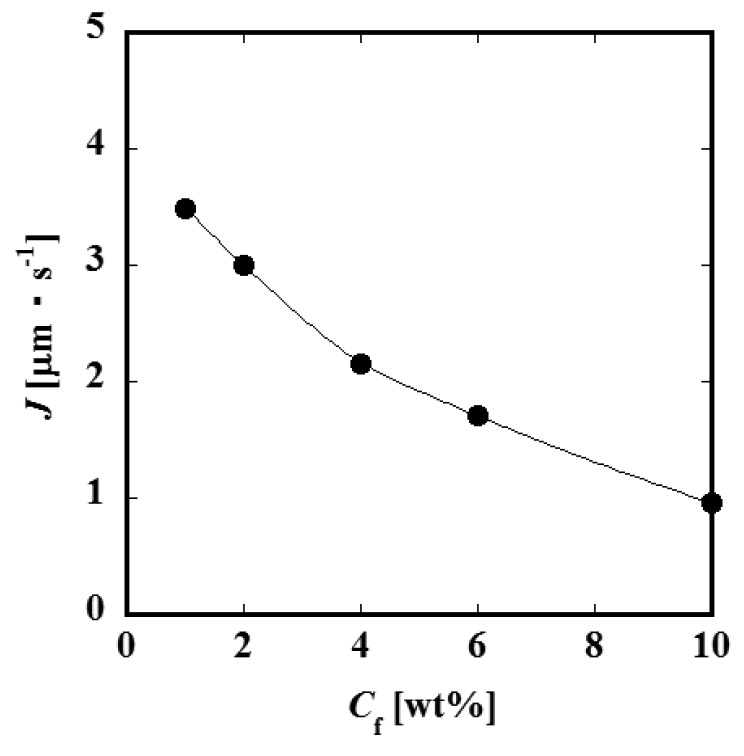
Relationship between permeate flux and feed liquid concentration (*L* = 60 mm, *Q*_f_ = 20 μL·min^−1^, *TMP* = 150 kPa).

Figure 4 (a) and Figure 4 (b) show the relationships between the constant permeate flux and *TMP* for *L* = 60 and 90 mm. It was observed that the permeate flux increases with feed liquid flow rate *Q*_f_. This result suggests that the convective mass transfer in the axial direction of the feed side channel would influence the permeate process significantly. The values of the permeate flux of the module of the channel length *L* = 90 mm are smaller than those of *L* = 60 mm. Considering that the feed liquid is more concentrated in a longer channel, it is expected that the difference in permeate flux between *L* = 60 and 90 mm would be attributed to the difference in concentration. Figure 5 shows the relationship between permeate flux and feed liquid concentration. The permeate flux decreases with an increase of the concentration. Based on this result, feed liquid concentration is considered to have some relationship with permeate flux as described above. With an increase of the feed liquid concentration, both the osmotic pressure difference between the feed and permeate side and viscosity of the feed liquid increase. Viscosity of the permeate liquid would not increase even if the feed liquid viscosity increases because the PVP is considered to be almost completely rejected by the membrane. The viscosity of the feed liquid, therefore, would not significantly affect the permeation through the membrane. The difference of osmotic pressure can be correlated with the concentration of PVP by the following experimental equation [7].

(2) $$\Pi =-1.23\times 10^{-2}C^{3}+5.83\times 10^{-1}C^{2}-7.61\times 10^{-2}C$$

where *∏* = 45.2 kPa is obtained when *C* = 10 wt% is substituted into this equation. This value is not negligible compared with the range of *TMP* = 75 to 150 kPa. Considering this result, the increase of the osmotic pressure difference with increase of the concentration of the feed liquid is expected to influence the permeate flux.

On the basis of the experimental results, a new model, considering the effect of the axial convection, the osmotic pressure difference and the concentration polarization, is developed in order to estimate the permeate flux of the ultrafiltration module.

## 4. A New Model of the Transport Phenomena in the Feed Side Channel and the Permeation

**Figure 6:**
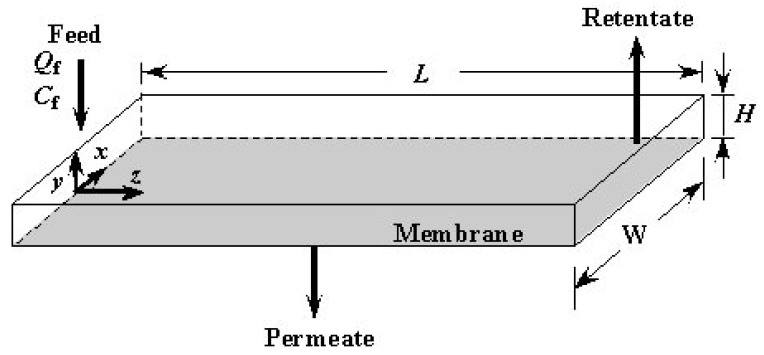
Schematic illustration of the feed side channel.

Figure 6 shows the geometry of the modeling of the feed side channel and the coordinate used in the model. Reynolds number in the channel is defined by the following equation.

(3) $$\text{Re}=\frac{\rho u_{\text{a}}d_{\text{e}}}{\mu }$$

where *u*_a_ is cross average velocity and *d*_e_ is equivalent diameter of the channel defined by the following equation.

(4) $$d_{\text{e}}=\frac{2\mathrm{HW}}{H+W}$$

**Figure 7:**
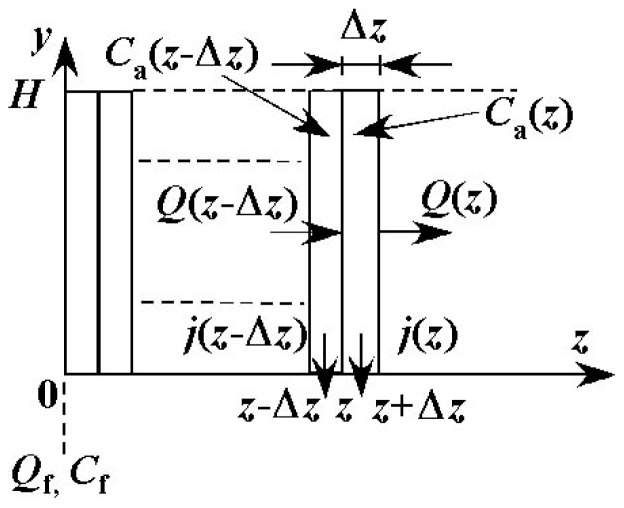
Control volumes in the feed side channel.

The value of Reynolds number (Re) is 2.08 under conditions where *Q*_f_ = 100μL·min^−1^ and *C*_f_ = 0 wt %, *i.e.* water without solute. In the range of the operational conditions of the experiments, the value of Re is less than 2.08 because *Q*_f_ is less than 100 μL·min^−1^ and viscosity of the feed liquid is larger than that of water. The flow in the channel is, therefore, considered to be laminar flow. In addition, the ratio of width to height *W*/*H* is 15, and this value is large enough such that the velocity, concentration and permeate flux are assumed to be uniform in the *x* direction. In the following, the distributions of physical quantities in the *y* and *z* directions only are considered. The channel is divided into very small control volumes in the *z* direction as shown in Figure 7. The width of each control volume Δ*z* is 5.00 × 10^−4^ m. Values of Δ*z*/*L* are 8.33 × 10^−3^ for *L* = 60 mm and 5.55 × 10^−3^ for *L* = 90 mm. The values are small enough such that the change in physical quantities within each control volume can be neglected. The entrance length is calculated by 0.065*d*_e_Re. The order of the value is 10^−2^ mm. It is much smaller than the length of the channel and the effect of the entrance on the flow field can be neglected.

In the model, it is assumed that the permeate liquid does not contain any solute. Accordingly, the concentration distribution is expressed as the following function of *y* and *z* based on the model of the concentration polarization.

(5) $$y<\delta (z):C(y,z)=C_{\text{f}}\text{exp}\left\{\left(1-\frac{y}{\delta (z)}\right)\text{ln}\frac{C(0,z)}{C_{\text{f}}}\right\}$$

(6) $$y\geq \delta (z):C(y,z)=C_{\text{f}}$$

where *C*(*y*,*z*) is the concentration at an arbitrary position in the channel and *C*(0,*z*) is the concentration at the membrane surface at *z* = *z*. *δ*(*z*) is the thickness of the concentration polarization layer calculated by the following equation.

(7) $$\delta (z)=\frac{D}{j(z)}\text{ln}\frac{C(0,z)}{C_{\text{f}}}$$

where *D* is the diffusion coefficient of PVP in water and *j*(*z*) is the local permeate flux at *z* = *z*. *δ*(*z*) increases in the *z* direction. In the downstream region where *δ*(*z*) > *H*, *y* is smaller than *δ*(*z*) in the whole cross section and the concentration is calculated by Equation (5). *j*(*z*) is considered to be expressed by the following equation.

(8) $$j(z)=(1-aC(0,z))\frac{\mathrm{TMP}(z)-\Pi (C(0,z))}{\mu R_{\text{M}}}$$

A deposit layer was hardly observed after the experiments. Only the membrane resistance *R*_M_ is, therefore, taken into account in the equation. The effect of the pore blockage, which is considered to depend on the concentration at the membrane surface *C*(0,*z*), should be considered. In the above equation, *aC*(0,*z*) represents the degree of pore blockage to first approximation. *a* is a fitting parameter. On the basis of the assumptions and the mass balance, the distributions of permeate flux under various conditions are calculated as follows.

In the following, the relationships among physical quantities *Q*, *C*, *C*_a_, *j*, *TMP*, *dP*/*dz u_z_*(*y*,*z*) and so on, in the control volumes at *z* = *z*−Δ*z* and *z* = *z*, are shown. The solvent permeates through the membrane in the control volume. The permeate flux should be taken into account in the mass balance of the feed liquid as follows.

(9) Q(z−Δz)−Q(z)=j(z)ΔzW

Considering that the solute PVP would not permeate through the membrane, the mass balance of the solute around the control volume at *z* = *z* is expressed as

(10) $$Q(z-\Delta z)C_{\text{a}}(z-\Delta z)=Q(z)C_{\text{a}}(z)=Q_{\text{f}}C_{\text{f}}$$

*Q*(*z*) is flow rate and *C*_a_(*z*) is the averaged concentration over the cross section at *z* = *z*. *TMP* at *z* = *z* is described as follows.

(11) $$\mathrm{TMP}(z)=\mathrm{TMP}(z-\Delta z)+{\frac{\mathrm{dP}}{\mathrm{dy}}|}_{z=z-\Delta z}\Delta z$$

Considering that the *y* component of velocity is much smaller than the *z* component *u_z_*(*y*,*z*), momentum balance at *y* = *y* in the control volume is expressed by the following equation.

(12) $$\frac{\partial }{\partial y}\left(\mu \frac{\partial u_{z}(y,z)}{\partial y}\right)={\frac{\mathrm{dP}}{\mathrm{dz}}|}_{z=z}$$

Viscosity *μ* is the function of *C*(*y*,*z*) in Equations (5) or (6) as described by Equation (1). The following function *α*(*y*,*z*) is defined.

(13) $$\alpha (y,z)=\frac{u_{z}(y,z)}{{\frac{\mathrm{dP}}{\mathrm{dz}}|}_{z=z}}$$

Considering that the pressure gradient in the *z* direction in the above equation is constant over the cross section, Equation (12) is, therefore, transformed to

(14) $$\frac{\partial }{\partial y}\left(\mu \frac{\partial \alpha (y,z)}{\partial y}\right)=1$$

*α*(*y*,*z*) is obtained as the result of the integration of Equation (14) under boundary conditions of *u_z_*(0,*z*) = *u_z_*(*H*,*z*) = 0. The following relationships among *u_z_*(*y*,*z*), *Q*(*z*), *C*_a_(*z*), *dP*/*dy*|*_z_*_=_*_z_* should be satisfied.

(15) $$Q(z)=W\int_{0}^{H}u_{z}(y,z)\mathrm{dy}=W\int_{0}^{H}\alpha (y,z)\mathrm{dy}\cdot {\frac{\mathrm{dP}}{\mathrm{dy}}|}_{z=z}$$

(16) $${\text{C}}_{\text{a}}(z)=\frac{W\int_{0}^{H}C(y,z)u_{z}(y,z)\mathrm{dy}}{Q(z)}=\frac{\int_{0}^{H}C(y,z)\alpha (y,z)\mathrm{dy}}{\int_{0}^{H}\alpha (y,z)\mathrm{dy}}$$

**Figure 8:**
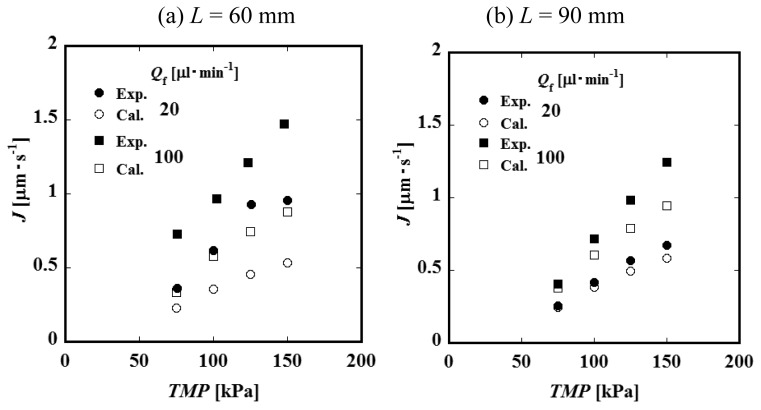
Comparisons of calculated and experimental results (*C*_f_ = 10 wt %) at (**a**) *L* = 60 mm, and (**b**) *L* = 90 mm.

Though the values of the physical quantities at *z* = *z* are fixed by the above equations under the condition that the values at *z* = *z* − Δ*z* are known, the relationships among the quantities are not explicit and the values cannot be calculated straightforwardly. The calculation was, therefore, carried out by a trial and error method as follows. *TMP*(*z*) was calculated by Equation (11). A value a little larger than the known *C*(0, *z* − Δ*z*) was assumed to be an initial temporal value of *C*(0,z) because the feed liquid at *z* = *z* was concentrated more than that at *z* − Δ*z*. By using the value, *j*(*z*), *C*(*y*,*z*), *Q*(*z*), *C*_a_(*z*), *α*(*y*,*z*), and *dP*/*dy*|*_z_*_=_*_z_*, are obtained sequentially by Equations (8), (5), (6), (9), (10), (14) and (15). After that, *C*_a_(*z*) was calculated again by Equation (16). Then, the value was compared with the value calculated by Equation (10). If both values did not coincide with each other within an acceptable error, another value of *C*(0,z) was assumed and the calculation described above was repeated until all values converged. Values of physical quantities in the control volume at the inlet were given as experimental conditions. By using them, the values of the quantities in the adjacent control volume were calculated. After that, the same calculations were made for sequential control volumes. As results, axial distributions of permeate flux in a module for various conditions were calculated. Values of averaged permeate flux *J* were obtained as averages of *j*(*z*) over the axial direction of the channel. Figure 8 (a) and Figure 8 (b) show the comparisons of calculated and experimental results. The fitting parameter *a* in Equation (8) was determined so as to correlate the results of *L* = 90 mm *Q*_f_ = 20 μL·min^−1^ appropriately to first approximation. The value of *a* was 1.00. The tendencies of the calculated results seem to agree with the experimental results. It would be suggested that the axial convection and the osmotic pressure difference have significant effects on the permeation process. On the other hand, the calculated values of *L* = 90 mm, *Q*_f_ = 100 μL·min^−1^ and *L* = 60 mm do not seem to agree well with the experimental ones. The differences are not negligible. In the calculation, *R*_M_ = 3.35 × 10^13^ m^−1^ was used as the membrane resistance. This value was obtained as an average of the resistances calculated by the results of the water flux for various experimental conditions. The values of the resistances were scattered. The standard deviation was larger than 40% of the average values. The reason for the large deviation was attributed not only to the difference in the resistance of each membrane itself but also to the differences caused by the fabrication, very small dust on the membrane, *etc.*, because the effective membrane area in the module was very small. In addition, the influence of *a* should be discussed. In the calculation, *a* was determined by using the results of *L* = 90 mm and *Q*_f_ = 20 μL·min^−1^. The difference between the experimental and calculated results of *L* = 60 mm are larger than those of *L* = 90 mm, *Q*_f_ = 100 μL·min^−1^. As described above, feed liquid is more concentrated in a longer channel. The concentration on the membrane surface would also be larger. In this model, the degree of pore blockage is represented by *aC*(0,*z*). If the degree would not be in proportion to *C*(0,*z*), *a* would change with the membrane surface concentration. The calculated values of *J* for *L* = 60 mm were smaller than the experimental ones. It is suggested that the value of *a* of *L* = 60 mm might be smaller than that of *L* = 90 mm, considering that permeate flux becomes small if a large value of *a* is used. Though the influence of *R*_M_ and the dependency of the degree of pore blockage on concentration are not clear at this stage, they should be discussed in the next stage.

## 5. Conclusions

Cross-flow ultrafiltration modules with microchannels, which are considered to be used for the filtration of macromolecular solution, were designed and fabricated. By means of the modules, experiments of ultrafiltration of PVP aqueous solution were carried out. From the results of the experiments, it appears that the flow rate, the concentration of feed liquid and the length of the channel have significant influence on permeate flux. Based on the results, the effects of the axial convection and the osmotic pressure difference are expected to be important factors in the permeation process. Considering these results, a new model taking the effects into account was developed and the values of averaged permeate flux were calculated. The tendencies of the calculated results agreed relatively well with the experimental ones. This would mean that the factors taken into account were appropriate. However, the absolute values of permeate flux did not agree well. As next steps, the influence of *R*_M_ and the dependency of the degree of pore blockage on the concentration should be discussed.
